# Supplementary material for: Phagocytosis of Primary Human Macrophages is Elevated by Ex Vivo Supplementation with n‐3 PUFA
Source: Mol Nutr Food Res. 2026 Apr 24;70:e70475. doi: 10.1002/mnfr.70475 (PMC13109673; doi:10.1002/mnfr.70475)
Supplement: Supplementary file 1 — Supporting File: mnfr70475‐sup‐0001‐SuppMat.pdf. [file MNFR-70-e70475-s001.pdf]

Phagocytosis of primary human macrophages is elevated by ex vivo supplementation with n-3 PUFA

Supplementary Material

Rebecca Kirchhoff, Michel André Chromik, Nils Helge Schebb*

Chair of Food Chemistry, School of Mathematics and Natural Sciences,
University of Wuppertal

***contact information of the corresponding author:**
Nils Helge Schebb
Chair of Food Chemistry, Faculty of Mathematics and Natural Sciences, University of Wuppertal
Gaussstrasse 20, 42119 Wuppertal
Email: nils@schebb-web.de
Phone: +49-202-439-3457

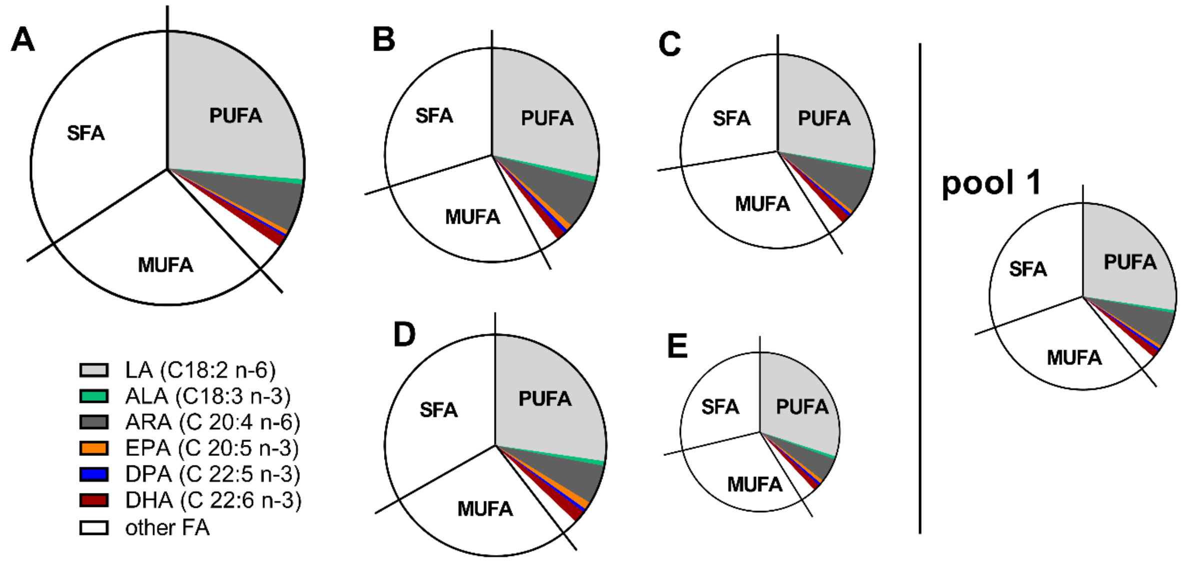

Figure S1: Relative fatty acid profiles of the tested human citrate plasmas. Non-fasting plasma of five subjects from a local blood donation center was analyzed for total FA concentrations by means of LC-MS/MS. Plasmas C and E were pooled resulting in plasma pool 1. Sizes of the circles indicate relative total fatty acid concentrations (plasma A, 11.0 ± 0.7 mM; plasma B, 8.7 ± 0.7 mM; plasma C, 7.8 ± 0.5 mM; plasma D, 9.0 ± 0.7 mM; plasma E, 6.4 ± 0.4 mM; pool 1, 7.5 ± 0.7 mM). SFA, saturated fatty acid; MUFA, monounsaturated fatty acid; PUFA, polyunsaturated fatty acid.

Table S1: Fatty acid pattern of the tested human citrate plasmas. Non-fasting plasma of five subjects (A-E) from a local blood donation center and plasma pool 1 (pooled plasma of C and E) was analyzed for total FA concentrations by means of LC-MS/MS. Results are shown as mean ± SD, $n = 3$. %n-6 in HUFA was calculated from fatty acid concentrations of C20:3 n-6, C20:4 n-6, C22:4 n-6, C22:5 n-6, C20:3 n-9, C20:5 n-3, C22:5 n-3, C22:6 n-3.

	**SFA** conc [mM]	**MUFA** conc [mM]	**HUFA** conc [mM]	**n-6 PUFA** conc [mM]	**n-3 PUFA** conc [mM]	**all FA** conc [mM]	**%n-6 in HUFA** [%]
**target**					<0.25	<10	>75
**A**	3.8 ± 0.2	3.0 ± 0.3	1.2 ± 0.1	3.8 ± 0.2	0.30 ± 0.02	11.0 ± 0.7	78.2 ± 0.5
**B**	2.6 ± 0.4	2.4 ± 0.2	1.1 ± 0.1	3.3 ± 0.2	0.34 ± 0.03	8.7 ± 0.7	75.4 ± 1.4
**C**	2.1 ± 0.1	2.4 ± 0.2	1.0 ± 0.1	2.9 ± 0.2	0.23 ± 0.01	7.8 ± 0.5	78.8 ± 1.4
**D**	3.0 ± 0.2	2.4 ± 0.2	1.0 ± 0.1	3.1 ± 0.2	0.37 ± 0.03	9.0 ± 0.7	67.3 ± 1.2
**E**	1.8 ± 0.2	1.9 ± 0.2	0.66 ± 0.03	2.4 ± 0.2	0.21 ± 0.02	6.4 ± 0.6	72.8 ± 1.4
**pool 1**	2.3 ± 0.3	2.3 ± 0.3	0.8 ± 0.1	2.7 ± 0.2	0.22 ± 0.02	7.5 ± 0.7	76.6 ± 1.3

Table S2: Oxidation status of the tested human citrate plasmas. Non-fasting plasma of five subjects (A-E) from a local blood donation center and plasma pool 1 (pooled plasma of C and E) was analyzed for total oxylipin concentrations by means of LC-MS/MS. Shown are concentrations of representative oxylipins formed by autoxidation and/or enzymatic activity as mean ± SD, *n* = 3.

	**target**	**plasma A**	**plasma B**	**plasma C**	**plasma D**	**plasma E**	**pool 1**
	[nM]	conc [nM]	conc [nM]	conc [nM]	conc [nM]	conc [nM]	conc [nM]
**5-HETE**	<100	17.6 ± 0.6	17.3 ± 0.9	13.6 ± 0.8	15 ± 1	9 ± 0.5	12 ± 1
**12-HETE**	<200	17 ± 1	22 ± 1	14.5 ± 0.6	13.3 ± 0.1	13 ± 1	15 ± 1
**15-HETE**	<200	21 ± 2	24 ± 2	16.8 ± 0.6	15.2 ± 0.6	9.5 ± 0.9	14.3 ± 0.9
**5-F2t-IsoP**	<1	0.43 ± 0.04	0.52 ± 0.07	0.21 ± 0.05	0.51 ± 0.12	0.31 ± 0.06	0.35 ± 0.03
**9-HODE**	<500	137 ± 9	137 ± 7	197 ± 13	214 ± 5	79 ± 7	137 ± 10

Table S3: Fatty acid status of erythrocytes from the human subjects who donated the monocytes used for n-3 PUFA supplementation experiments. Erythrocytes were analyzed for total fatty acid concentrations by means of LC-MS/MS and %n-6 in HUFA was calculated from fatty acid concentrations of C20:3 n-6, C20:4 n-6, C22:4 n-6, C22:5 n-6, C20:3 n-9, C20:5 n-3, C22:5 n-3, C22:6 n-3. All donors were healthy, voluntary blood donors from local blood donation centers. Results are shown as mean ± SD, *n* = 3.

**experi-ment**	**subject**	**%n-6 in HUFA** [%]	**LA** (C18:2 n-6) conc [µM]	**ARA** (C20:4 n-6) conc [µM]	**EPA** (C20:5 n-3) conc [µM]	**DHA** (C22:6 n-3) conc [µM]	**DPA** (C22:5 n-3) conc [µM]
1	1	76.8 ± 0.6	626 ± 93	699 ± 48	36 ± 3	148 ± 16	112 ± 11
	2	74.6 ± 0.6	914 ± 184	745 ± 93	31 ± 5	188 ± 31	116 ± 17
	3	74.7 ± 0.4	538 ± 20	842 ± 56	37 ± 2	207 ± 9	135 ± 5
	4	71.1 ± 0.3	616 ± 40	683 ± 47	35 ± 3	221 ± 12	113 ± 6
2	5	75.4 ± 1.0	764 ± 29	674 ± 23	26 ± 3	222 ± 24	86 ± 6
	6	70.0 ± 0.6	844 ± 41	585 ± 45	50 ± 3	220 ± 9	106 ± 5
	7	76.2 ± 0.3	751 ± 4	709 ± 17	31 ± 1	187 ± 4	105 ± 5
	8	75.9 ± 0.6	1322 ± 117	753 ± 86	44 ± 4	166 ± 15	136 ± 7

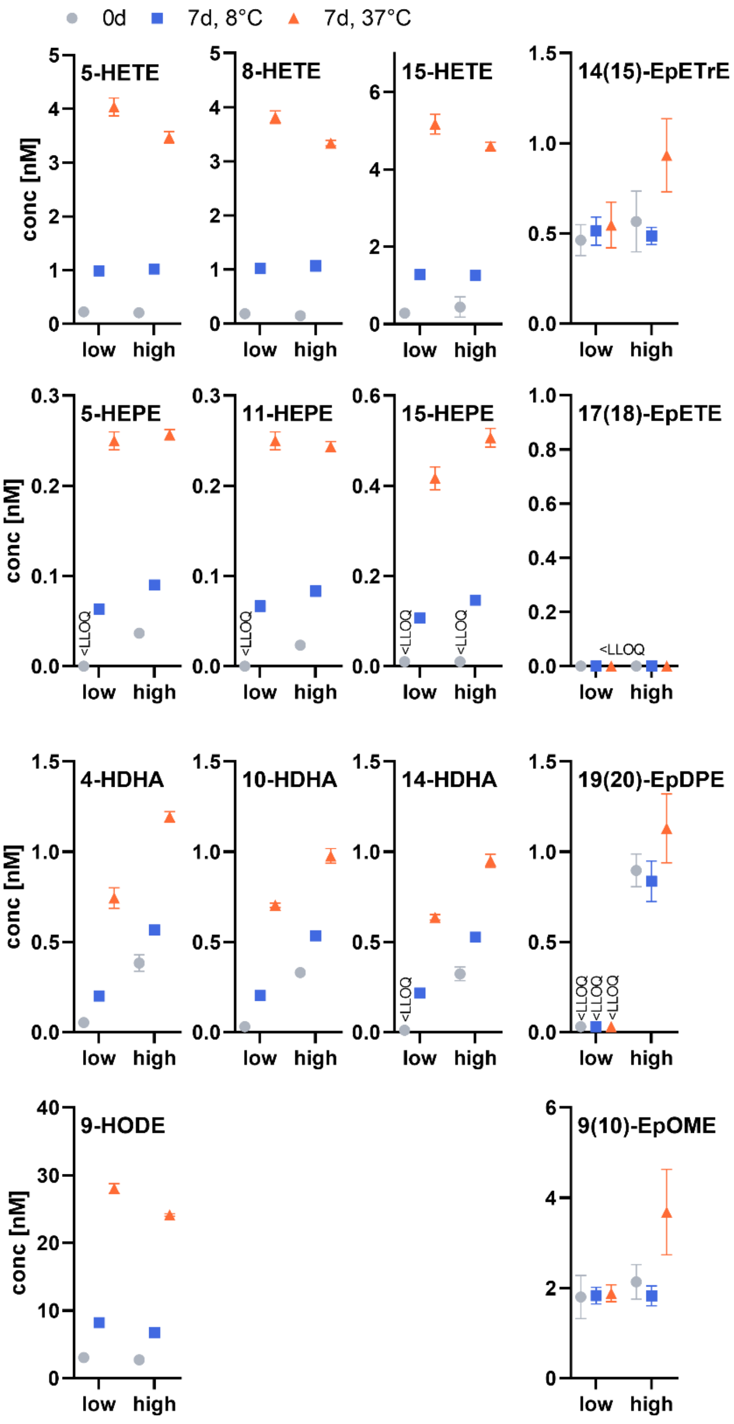

Figure S2: Oxylipin concentrations of the used cell culture media at baseline and after storage for 7 days. Media were prepared with 5% (*v*/*v*) plasma pool 1 (non-supplemented medium, low). For the supplemented medium (high), additionally 10.3 µM DHA (>99%) and 5.5 µM EPA (>99%) were added. Concentrations of selected, most abundant oxylipins derived from ARA, EPA, DHA and LA were analyzed at beginning and after 7 days of incubation at 8 °C or 37 °C by means of LC-MS/MS. Results of one representative experiment are shown as mean ± SD, $n = 3$.

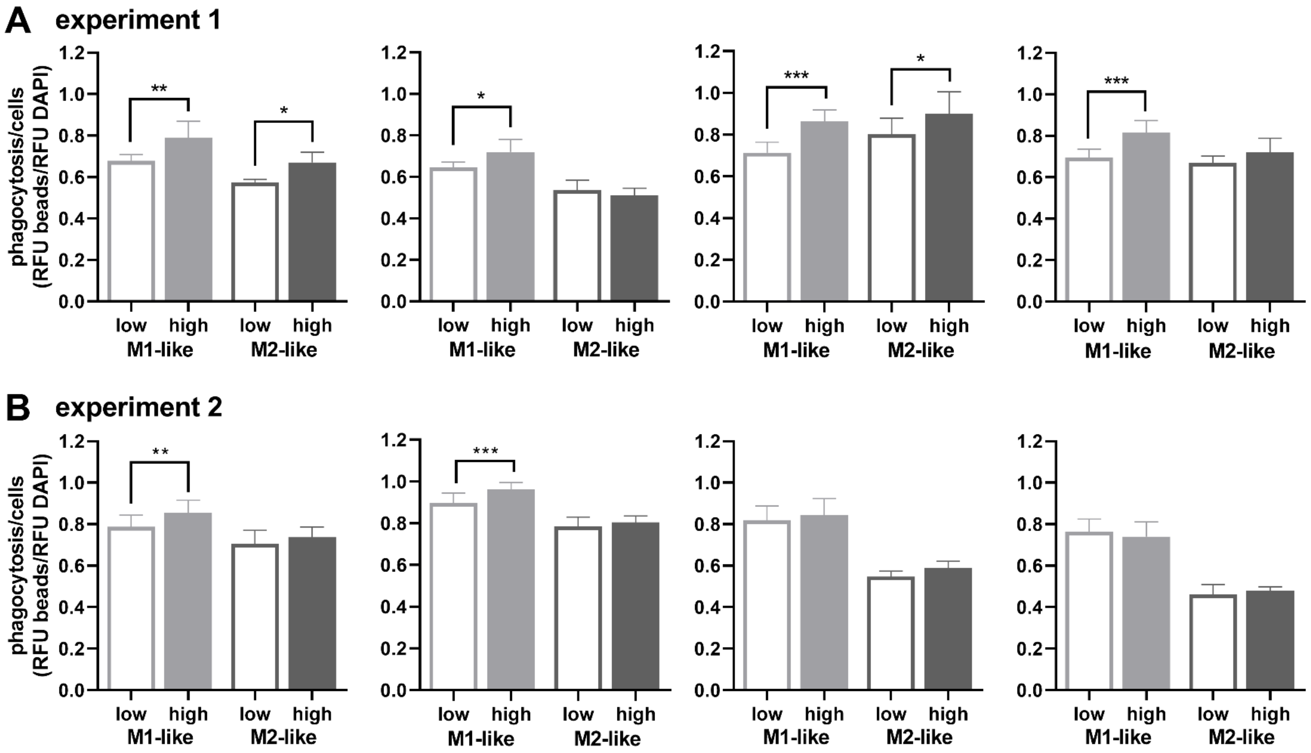

Figure S3: Impact of n-3 PUFA supplementation on phagocytosis in the two independent supplementation experiments (A, B). After isolation of monocytes, cells were differentiated into macrophages and supplemented using medium with 5% (*v*/*v*) plasma pool 1 (low) or plasma pool 1 and 10.3 µM DHA (>99%) and 5.5 µM EPA (>99%) (high) for 2 days. Cells were pooled transferred into 96 well plates (50,000 cells per well) for phagocytosis assay. Shown is phagocytosis as ratio of bead fluorescence to DAPI fluorescence. Results of the four 96 well plates for each supplementation experiment are shown as mean ± SD for *n* = 5-8 (A) or *n* = 14-15 (B) replicates from a pool of 4 subjects. Statistical analysis was performed by 1-way ANOVA followed by Sidak's multiple comparison test. Differences from vehicle control were considered significant at *p* values ≤0.5 (*), ≤0.01 (**) or ≤0.001 (***).

Table S4: Concentrations of oxylipin standards (stock solutions) used for supplementation of the medium analyzed by means of LC-MS/MS.

mix	analyte	concentration nominal [mM]	concentration measured [mM]
I	4-HDHA	0.29	0.25
	7-HDHA	0.29	0.32
	5-HEPE	0.31	0.18
II	14(*S*)-HDHA	0.29	0.21
	17(*S*)-HDHA	0.29	0.22
	12(*S*)-HEPE	0.31	0.41
	15(*S*)-HEPE	0.31	0.42
III	19(20)-EpDPE	0.29	0.28
	17(18)-EpETE	0.31	0.43

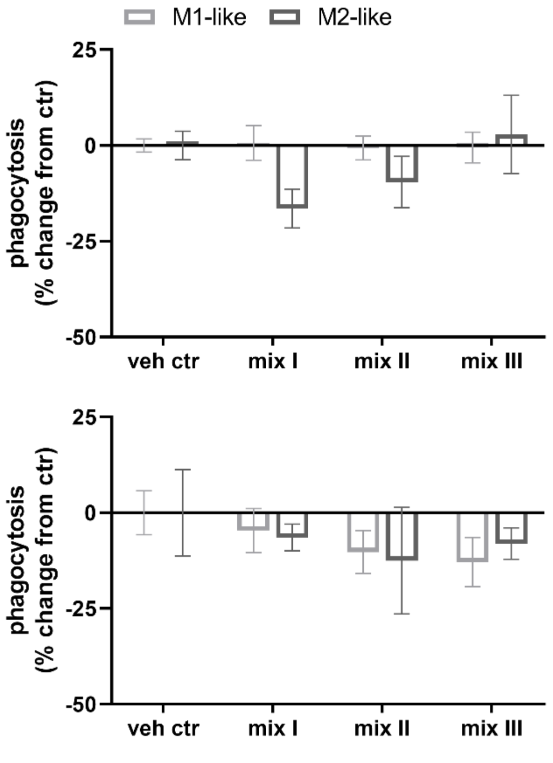

Figure S4: Impact of mixtures of oxylipins on phagocytosis. Non-supplemented macrophages were transferred into 96 well plates (50,000 cells per well) and incubated with 300 nM oxylipins (mix I: 5-HEPE, 4-HDHA, 7-HDHA; mix II: 12(*S*)-, 15(*S*)-HEPE, 14(*S*)-, 17(*S*)-HDHA; mix III: 17(18)-EpETE, 19(20)-EpDPE, Table S4) or 0.1% DMSO (veh ctr) for 1 h. Shown are the results of two 96 well plates as % change from vehicle control as mean ± SD for *n* = 4-5 replicates from a pool of 4 subjects.

Table S5: Concentrations of added oxylipins in the media after phagocytosis assay. Macrophages were preincubated with a mix of oxylipins (300 nM) for 1 h. Phagocytosis was started by addition of fluorescent beads and the mix of oxylipins was renewed. After 2 h the medium was collected and analyzed for non-esterified oxylipins by means of LC-MS/MS. Results of one representative experiment are shown as mean ± SD, *n* = 3.

**mix**	**oxylipin**	**M1-like conc** [nM]			**M2-like conc** [nM]		
**I**	5-HEPE	246	±	10	173	±	7
	4-HDHA	162	±	6	124	±	9
	7-HDHA	189	±	5	161	±	8
**II**	12-HEPE	125	±	1	105	±	4
	15-HEPE	143	±	2	115	±	3
	14-HDHA	128	±	4	110	±	3
	17-HDHA	204	±	1	158	±	7
**III**	17(18)-EpETE	232	±	8	159	±	7
	17,18-DiHETE	18	±	1	26	±	2
	19(20)-EpDPE	294	±	13	238	±	5
	19,20-DiHDPE	14	±	1	21	±	1

Table S6: Concentrations of added oxylipins in the media after phagocytosis assay. Macrophages were preincubated with 100 nM PGE_2 (control) or additionally a mix of oxylipins (300 nM) for 1 h. Phagocytosis was started by addition of fluorescent beads and the mix of oxylipins was renewed. After 2 h the medium was collected and analyzed for non-esterified oxylipins by means of LC-MS/MS. Results of one representative experiment are shown as mean ± SD, *n* = 3.

**mix**	**oxylipin**	**M1-like conc** [nM]			**M2-like conc** [nM]		
control	PGE_2	90	±	2	96	±	1
**I**	5-HEPE	12.5	±	0.4	21	±	1
	4-HDHA	10	±	1	14	±	1
	7-HDHA	12	±	1	17	±	1
	PGE_2	86	±	4	88	±	2
**II**	12-HEPE	7.9	±	0.4	10	±	1
	15-HEPE	8.9	±	0.4	12	±	1
	14-HDHA	8.2	±	0.4	10	±	1
	17-HDHA	14	±	2	21	±	2
	PGE_2	84	±	2	91	±	2
**III**	17(18)-EpETE	17	±	1	22	±	2
	17,18-DiHETE	4.1	±	0.1	2.8	±	0.2
	19(20)-EpDPE	25	±	1	29	±	1
	19,20-DiHDPE	3.0	±	0.1	1.7	±	0.2
	PGE_2	87	±	5	92	±	9

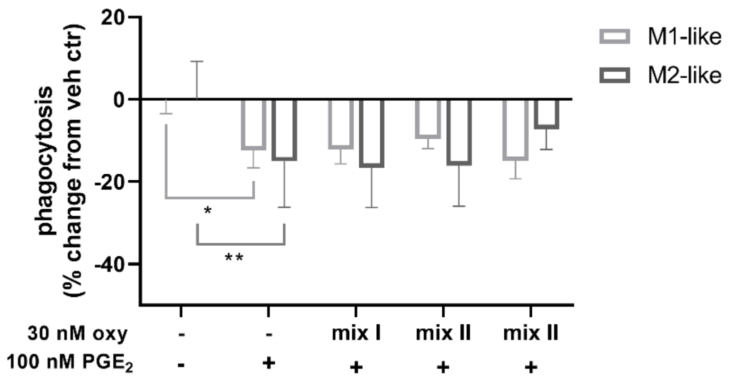

Figure S5: Impact of n-3 PUFA derived oxylipins on the inhibitory effect of PGE_2 on phagocytosis. Primary human macrophages were preincubated with 100 nM PGE_2 and 30 nM oxylipins (mix I: 5-HEPE, 4-HDHA, 7-HDHA; mix II: 12(*S*)-, 15(*S*)-HEPE, 14(*S*)-, 17(*S*)-HDHA; mix III: 17(18)-EpETE, 19(20)-EpDPE, Table S4) or 0.1% DMSO (veh ctr) for 1 h, followed by phagocytosis for 2 h. Shown is phagocytosis as % change from vehicle control as mean ± SD for $n = 4$-5 replicates from a pool of 4 subjects. Statistical analysis was performed by 1-way ANOVA followed by Sidak's multiple comparison test. Differences from vehicle control were considered significant at p values ≤0.5 (*), ≤0.01 (**).

Table S7: Statistical analysis of changes in A) %n-6 in HUFA, B) relative FA pattern and C) relative PUFA pattern in macrophages after supplementation with n-3 PUFA. Statistical differences were determined using A) unpaired t-test or B-C) two-way ANOVA followed by Sidak's multiple comparisons test.

A)

experiment	low vs high	t	df	*p* value	
1	M1-like	20.98	4	*<0.001*	***
	M2-like	12.57	4	*<0.001*	***
2	M1-like	60.98	4	*<0.001*	***
	M2-like	40.49	4	*<0.001*	***

B)

experiment			mean difference	95% CI of difference	adjusted *p* value	
1	low M1 vs	SFA	-0.30	-3.78 to 3.18	*>0.05*	*ns*
	high M1	MUFA	1.52	-1.96 to 5.00	*>0.05*	*ns*
		n-6 PUFA	2.62	-0.86 to 6.10	*>0.05*	*ns*
		n-3 PUFA	-3.91	-7.39 to -0.43	0.024	*
1	low M2 vs	SFA	-2.29	-5.66 to 1.07	*>0.05*	*ns*
	high M2	MUFA	3.98	0.61 to 7.35	0.017	*
		n-6 PUFA	2.93	-0.43 to 6.31	*>0.05*	*ns*
		n-3 PUFA	-4.65	-8.02 to -1.28	0.005	**
2	low M1 vs	SFA	4.33	-0.04 to 8.70	*>0.05*	*ns*
	high M1	MUFA	1.30	-3.07 to 5.67	*>0.05*	*ns*
		n-6 PUFA	1.23	-3.14 to 5.61	*>0.05*	*ns*
		n-3 PUFA	-6.97	-11.3 to -2.60	0.002	**
2	low M2 vs	SFA	2.20	-3.40 to 7.79	*>0.05*	*ns*
	high M2	MUFA	1.53	-4.06 to 7.12	*>0.05*	*ns*
		n-6 PUFA	2.94	-2.65 to 8.53	*>0.05*	*ns*
		n-3 PUFA	-6.73	-12.3 to -1.13	0.015	*

C)

experiment			mean difference	95% CI of difference	adjusted *p* value	
1	low M1	other n-6	11.1	9.54 to 12.6	*<0.001*	***
	vs high	LA (C 18:2 n-6)	-1.29	-2.82 to 0.23	*>0.05*	*ns*
	M1	ARA (C 20:4 n-6)	3.66	2.14 to 5.21	*<0.001*	***
		other n-3	-0.27	-1.79 to 1.26	*>0.05*	*ns*
		EPA (C 20:5 n-3)	-0.59	-2.12 to 0.94	*>0.05*	*ns*
		DPA (C 22:5 n-3)	-0.87	-2.40 to 0.66	*>0.05*	*ns*
		DHA (C 22:6 n-3)	-12.0	-13.5 to -10.4	*<0.001*	***
1	low M2	other n-6	9.87	7.44 to 12.3	<0.001	***
	vs high	LA (C 18:2 n-6)	0.43	-2.00 to 2.85	>0.05	*ns*
	M2	ARA (C 20:4 n-6)	4.80	2.38 to 7.22	<0.001	***
		other n-3	-0.13	-2.55 to 2.29	>0.05	*ns*
		EPA (C 20:5 n-3)	-0.85	-3.28 to 1.57	>0.05	*ns*
		DPA (C 22:5 n-3)	-0.7	-3.12 to 1.73	>0.05	*ns*
		DHA (C 22:6 n-3)	-13.6	-16.0 to -11.1	*<0.001*	***
2	low M1	other n-6	10.7	8.17 to 13.2	*<0.001*	***
	vs high	LA (C 18:2 n-6)	-0.17	-2.67 to 2.33	*>0.05*	*ns*
	M1	ARA (C 20:4 n-6)	8.45	5.95 to 11.0	*<0.001*	***
		other n-3	-0.28	-2.79 to 2.22	*>0.05*	*ns*
		EPA (C 20:5 n-3)	-1.87	-4.38 to 0.63	*>0.05*	*ns*
		DPA (C 22:5 n-3)	-5.86	-8.36 to -3.36	*<0.001*	***
		DHA (C 22:6 n-3)	-11.3	-13.8 to -8.82	*<0.001*	***
2	low M2	other n-6	9.29	6.89 to 11.7	*<0.001*	***
	vs high	LA (C 18:2 n-6)	3.26	0.86 to 5.66	0.004	**
	M2	ARA (C 20:4 n-6)	6.99	4.59 to 9.39	*<0.001*	***
		other n-3	-0.28	-2.69 to 2.12	*>0.05*	*ns*
		EPA (C 20:5 n-3)	-2.88	-5.28 to -0.48	0.012	*
		DPA (C 22:5 n-3)	-6.02	-8.42 to -3.62	*<0.001*	***
		DHA (C 22:6 n-3)	-10.6	-13.0 to -8.17	*<0.001*	***

Table S8: Statistical analysis of changes in the oxylipin pattern of human macrophages with and without supplementation with n-3 PUFA. Statistical differences were determined using unpaired t-test.

oxylipin	experiment	low vs high	t	df	*p* value	
4-HDHA	1	M1-like	8.12	4	0.0012	**
		M2-like	11.6	4	*<0.001*	***
	2	M1-like	11.2	4	*<0.001*	***
		M2-like	34.8	4	*<0.001*	***
7-HDHA	1	M1-like	4.38	4	0.012	*
		M2-like	4.89	4	0.008	*
	2	M1-like	17.9	4	*<0.001*	***
		M2-like	10.2	4	*<0.001*	***
14-HDHA	1	M1-like	7.25	4	0.002	**
		M2-like	8.22	4	0.001	**
	2	M1-like	12.9	4	*<0.001*	***
		M2-like	4.33	4	0.012	*
17-HDHA	1	M2-like	8.19	4	0.001	*
	2	M2-like	53.0	4	*<0.001*	***
19(20)-EpDPE	1	M1-like	25.5	4	*<0.001*	***
		M2-like	11.6	4	*<0.001*	***
	2	M1-like	22.8	4	*<0.001*	***
		M2-like	8.33	4	0.001	**
5-HEPE	1	M1-like	4.46	4	0.011	*
		M2-like	6.70	4	0.003	**
	2	M1-like	23.5	4	*<0.001*	***
		M2-like	42.2	4	*<0.001*	***
12-HEPE	2	M1-like	17.3	4	*<0.001*	***
		M2-like	11.3	4	*<0.001*	***
15-HEPE	1	M2-like	0.35	4	*>0.05*	*ns*
	2	M2-like	4.31	4	0.013	*
17(18)-EpETE	1	M1-like	31.1	4	*<0.001*	***
		M2-like	38.8	4	*<0.001*	***
	2	M1-like	10.7	4	*<0.001*	***
		M2-like	10.9	4	*<0.001*	***
5-HETE	1	M1-like	4.04	4	0.016	*
		M2-like	2.18	4	*>0.05*	*ns*
	2	M1-like	3.65	4	0.220	*
		M2-like	5.51	4	0.005	**
11-HETE	1	M1-like	18.4	4	*<0.001*	***
		M2-like	11.0	4	*<0.001*	***
	2	M1-like	9.05	4	*<0.001*	***
		M2-like	13.7	4	*<0.001*	***
12-HETE	1	M1-like	3.17	4	0.340	*
		M2-like	7.39	4	0.002	**
	2	M1-like	2.87	4	0.045	*
		M2-like	5.75	4	0.005	**

Table S8 continued.

oxylipin	experiment	low vs high	t	df	*p* value	
15-HETE	1	M1-like	2.55	4	*>0.05*	*ns*
		M2-like	15.5	4	*<0.001*	***
	2	M1-like	2.86	4	0.046	*
		M2-like	7.05	4	0.002	**
14(15)-EpETrE	1	M1-like	6.01	4	0.004	**
		M2-like	5.34	4	0.006	**
	2	M1-like	8.91	4	*<0.001*	***
		M2-like	7.86	4	0.001	**
12-HHTrE	1	M1-like	9.92	4	*<0.001*	***
		M2-like	18.7	4	*<0.001*	***
	2	M1-like	8.59	4	0.001	**
		M2-like	5.76	4	0.005	**
PGE_2	1	M1-like	1.28	4	*>0.05*	*ns*
		M2-like	1.43	4	*>0.05*	*ns*
	2	M1-like	3.02	4	0.040	*
		M2-like	6.50	4	0.003	**
PGD_2	1	M1-like	1.00	4	*>0.05*	*ns*
		M2-like	4.56	4	0.010	*
	2	M1-like	1.94	4	*>0.05*	*ns*
		M2-like	3.55	4	0.024	*
PGF_{2a}	1	M1-like	5.73	4	0.005	**
		M2-like	1.49	4	*>0.05*	*ns*
	2	M1-like	2.89	4	0.045	*
		M2-like	1.65	4	*>0.05*	*ns*
TxB_2	1	M1-like	15.3	4	*<0.001*	***
		M2-like	62.5	4	*<0.001*	***
	2	M1-like	8.59	4	0.001	**
		M2-like	5.78	4	0.004	**
